# Biomechanical Thresholds Regulate Inflammation through the NF-κB Pathway: Experiments and Modeling

**DOI:** 10.1371/journal.pone.0005262

**Published:** 2009-04-16

**Authors:** Jin Nam, Baltazar D. Aguda, Bjoern Rath, Sudha Agarwal

**Affiliations:** 1 Biomechanics and Tissue Engineering Laboratory, College of Dentistry, The Ohio State University, Columbus, Ohio, United States of America; 2 Mathematical Biosciences Institute, The Ohio State University, Columbus, Ohio, United States of America; 3 Center for Critical Care, The Ohio State University Medical Center, Columbus, Ohio, United States of America; 4 Orthopaedic Surgery, University of Regensburg, Regensburg, Germany; Hopital Cochin, France

## Abstract

**Background:**

During normal physical activities cartilage experiences dynamic compressive forces that are essential to maintain cartilage integrity. However, at non-physiologic levels these signals can induce inflammation and initiate cartilage destruction. Here, by examining the pro-inflammatory signaling networks, we developed a mathematical model to show the magnitude-dependent regulation of chondrocytic responses by compressive forces.

**Methodology/Principal Findings:**

Chondrocytic cells grown in 3-D scaffolds were subjected to various magnitudes of dynamic compressive strain (DCS), and the regulation of pro-inflammatory gene expression via activation of nuclear factor-kappa B (NF-κB) signaling cascade examined. Experimental evidences provide the existence of a threshold in the magnitude of DCS that regulates the mRNA expression of nitric oxide synthase (*NOS2*), an inducible pro-inflammatory enzyme. Interestingly, below this threshold, DCS inhibits the interleukin-1β (IL-1β)-induced pro-inflammatory gene expression, with the degree of suppression depending on the magnitude of DCS. This suppression of *NOS2* by DCS correlates with the attenuation of the NF-κB signaling pathway as measured by IL-1β-induced phosphorylation of the inhibitor of kappa B (IκB)-α, degradation of IκB-α and IκB-β, and subsequent nuclear translocation of NF-κB p65. A mathematical model developed to understand the complex dynamics of the system predicts two thresholds in the magnitudes of DCS, one for the inhibition of IL-1β-induced expression of *NOS2* by DCS at low magnitudes, and second for the DCS-induced expression of *NOS2* at higher magnitudes.

**Conclusions/Significance:**

Experimental and computational results indicate that biomechanical signals suppress and induce inflammation at critical thresholds through activation/suppression of the NF-κB signaling pathway. These thresholds arise due to the bistable behavior of the networks originating from the positive feedback loop between NF-κB and its target genes. These findings lay initial groundwork for the identification of the thresholds in physical activities that can differentiate its favorable actions from its unfavorable consequences on joints.

## Introduction

Exercise is essential for maintaining the health of cartilage, and is believed to have therapeutic effects on the degenerating cartilages in diseases like osteoarthritis [1], [2]. In addition, continuous passive motion (CPM) has been shown to allay pain and limited mobility due to the disease [3], [4]. Excessive exercise, however, could induce inflammation by itself that promotes damage of cartilage and aggravates the disease [5]. This double-edged sword is an intriguing phenomenon and its understanding has important medical significance. Identification of the threshold in exercise that delineates its favorable from its unfavorable consequences is a key issue being addressed in our laboratory. In this paper, we describe our experimental investigations describing the consequences of mechanical signals applied to chondrocytic cells, and demonstrate the existence of a threshold governing the expression of pro-inflammatory genes. In addition, we present a kinetic model of intracellular networks, and show that the model explains our experiments in ways that could not have been possible in the absence of an integrative mechanistic model.

Exercise generates biomechanical signals that are sensed by chondrocytes which then respond by adjusting their metabolic activities, including expression of genes that regulate inflammation. Chondrocytes perceive these signals, likely through cell surface receptors such as β-integrin and focal adhesion complexes [6], [7]. How these mechanical signals interface with the molecular regulatory pathways of a cell is a crucial question that we and other groups have attempted to answer recently [8], [9], [10]. Indeed, we have shown that the protein complex IκB kinase (IKK) is a key mediator of mechanical signals applied to chondrocytes [8], [10]. This observation establishes the link between the mechanical signals and the pathway of NF-κB activation, a family of dimeric transcription factors that regulate the expression of over two hundred genes, many of which are anti- and pro-inflammatory genes. Detailed biochemical mechanisms of this pathway can be found in an excellent review done by Hoffmann and Ghosh [11].

To apply biomechanical compressive forces to cells *in vitro*, similar to those experienced by chondrocytes in cartilage, it is necessary to embed cells in a scaffold which can efficiently transmit applied forces. In this study, we have used well-characterized biomechanically active scaffolds in which cultured cells can perceive and distinguish different magnitudes of dynamic compressive forces, and can respond to them accordingly [12], [13]. The differential responses of the cells from experiments were analyzed by linking to the NF-κB signaling pathway. The details of the NF-κB network are discussed in the modeling section of this paper. The network is very complex and mere intuitive reasoning is not sufficient to understand its behavior. This complex network exhibits various feedback loops, both negative and positive. Our mathematical analysis of the kinetic model will show that the positive feedback loops (i.e., the autocrine loops between NF-κB and pro-inflammatory cytokines) in the network are candidates for the mechanistic origins of the thresholds observed experimentally.

## Results and Discussion

**Figure 1:**
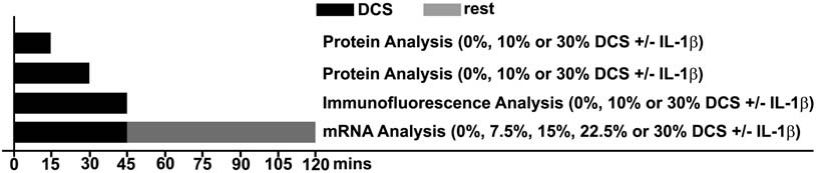
Schematic illustration of experimental regimens.

The well characterized electrospun scaffolds were employed in this study to apply different magnitudes of DCS to the embedded cells. Chondrocytic cells differentiated from mesenchymal cell line, C3H10T1/2, were utilized in all experiments (please see Figure S1 for details). Following 48 hours of cultivation, the cell-scaffold constructs were subjected to various magnitudes of DCS at 1 Hz in the absence or presence of recombinant human IL-1β. The regimens used in this study are shown in Figure 1.

### DCS inhibits or induces pro-inflammatory gene expression in a magnitude-dependent manner

**Figure 2:**
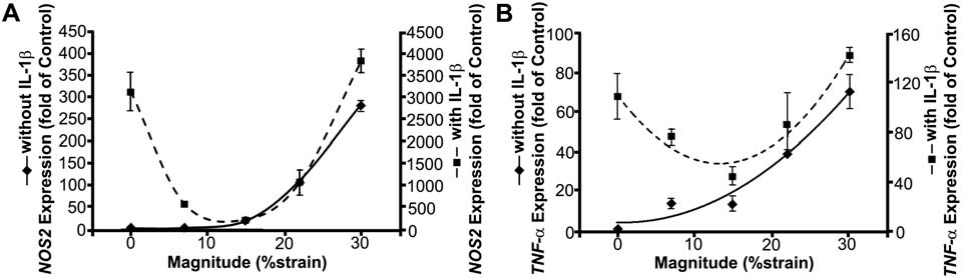
Regulation of pro-inflammatory gene expression by DCS. (A) *NOS2* and (B) *TNF-α* expression in the cells subjected to various magnitudes of DCS for 45 minutes followed by 75 minutes of rest, in the absence (solid line) or presence (dotted line) of exogenous IL-1β (2 ng/ml). The figure shows that in the absence of IL-1β, the pro-inflammatory gene expression markedly increased after a threshold of the applied DCS, whereas in the presence of IL-1β, the gene expression was suppressed in response to the DCS up to a threshold, followed by an increase proportional to the magnitude of applied DCS.

To examine how the magnitude of DCS differentially influences cellular responses, the gene expression of *NOS2*, one of the early-responsive pro-inflammatory genes was investigated. In the absence of inflammatory stimulus by IL-1β, the applied DCS induced *NOS2* gene expression depending on the magnitude of applied forces (solid curve in Fig. 2A). At low magnitudes, the cells did not respond to the applied DCS. However, the *NOS2* induction increased proportional to the applied DCS after a certain threshold, resulting in an approximately 300-fold increase at 30% DCS. On the other hand, exogenous IL-1β induced approximately 3000-fold increase in the *NOS2* expression (dotted curve in Fig. 2A). Interestingly, the IL-1β-induced gene upregulation was gradually suppressed by the application of DCS up to a threshold, which nearly coincides with the magnitude that initiated *NOS2* induction in the absence of the inflammatory cytokine. The gene response was parabolically related to the applied DCS having approximately 10–15% DCS estimated to result in the greatest suppression on IL-1β-induced *NOS2* gene expression (dotted curve in Fig. 2A). High magnitudes of DCS were ineffective in suppressing the IL-1β-induced *NOS2* gene expression; rather, they intensified the gene expression resulting in approximately 3800-fold increase at 30% DCS. Similar pro-inflammatory gene regulation by DCS was observed in *TNF-α* (Fig. 2B).

### DCS differentially regulates protein phosphorylation and degradation in the NF-κB signaling cascade depending on its magnitudes

**Figure 3:**
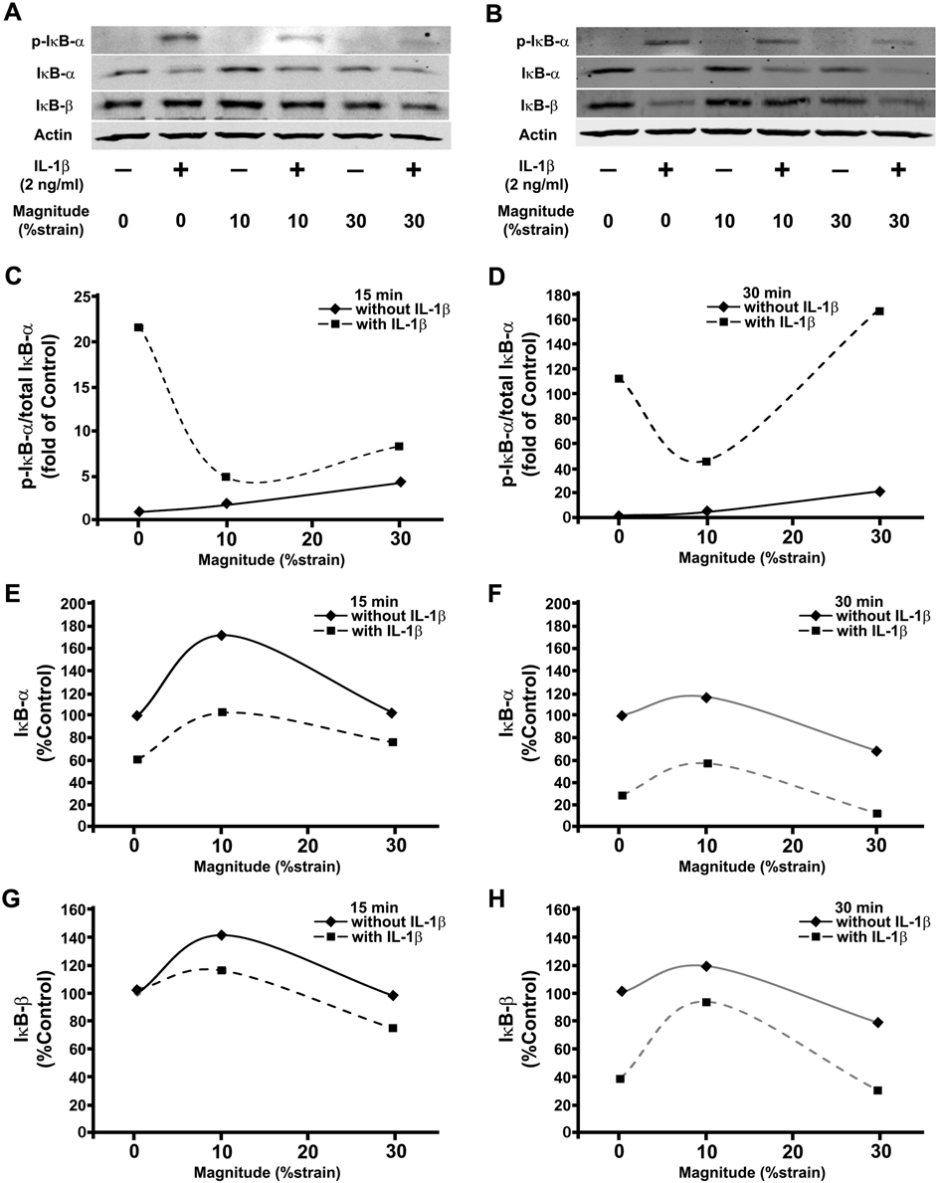
Regulation of IκB-α phosphorylation, and IκB-α and IκB-β degradation by DCS. Western blot analysis shows that 10% DCS inhibits and 30% DCS upregulates IL-1β-induced IκB-α phosphorylation and degradation follwoing 15 min (A) and 30 min (B) of activation. The blots were densitometrically analyzed for phospho-IκB-α/total IκB-α (C and D), IκB-α (E and F), and IκB-β (G and H) in the cells subjected to 0, 10 or 30% DCS in the absence or presence of IL-1β (2 ng/ml) for 15 minutes (C, E, G) or 30 minutes (D, F, H).

Based on the differential regulation of *NOS2* and *TNF-α* gene by DCS, the end products controlled by the NF-κB signaling pathway, the phosphorylation and degradation of proteins that regulate the NF-κB activity were investigated (Fig. 3). To confirm the biphasic behavior of DCS in each step of the NF-κB signal cascade, DCSs of 10% and 30% were chosen to represent physiological (anti-inflammatory) and hyper-physiological (pro-inflammatory) levels of stimulation, respectively, based on the inflammatory gene regulation (Fig. 2). IKK activity was estimated by monitoring IκB-α phosphorylation relative to total IκB-α at 15 or 30 minutes (Fig. 3C and 3D). In the absence of the stimulant, the phosphorylation ratio of IκB-α (phospho-IκB-α/total-IκB-α) was proportionally related to the applied DCS at both time points. However, the ratio increased more rapidly at the high magnitude (5-fold increase in 30% from 15 to 30 minutes versus 2-fold increase in 10%). When the cells were subjected to DCS and IL-1β simultaneously, the biomechanical stimulation attenuated the cytokine-induced IκB-α phosphorylation. Interestingly, the suppression of the phosphorylation was observed even in 30% at 15 minutes. However, at 30 minutes, the high magnitude induced higher phosphorylation ratio than IL-1β-treated samples without biomechanical stimulation.

Since the phosphorylation of IκB-α directly regulates the degradation of the protein by ubiquitination and proteasomal degradation, the changes in IκB-α were examined (Fig. 3E and 3F). At 15 minutes, IL-1β induced approximately 40% degradation of IκB-α, and this degradation was inhibited by the application of 10% DCS, almost to a similar level as untreated control. High magnitude also seems to suppress the protein degradation agreeing with the phosphorylation result at this early time point. Interestingly, 10% DCS seem to induce IκB-α synthesis resulting in approximately 80% increase in the protein amount (Fig. 3E). Longer treatment time (30 minutes) resulted in larger IκB-α degradation in IL-1β-treated sample, and the degradation was attenuated in the 10% DCS in the presence of the cytokine (Fig. 3F). Correlated to the phosphorylation result at 30 minutes, 30% DCS further intensified the degradation of IκB-α leading to less remaining protein compared to IL-1β treated sample without mechanical stimulation. Furthermore, it was observed that high magnitude of DCS alone could induce IκB-α degradation compared to low magnitude that maintains the protein level similar to untreated control. This may suggest synergistic effect between inflammatory cytokine and hyper-physiological force to accelerate the NF-κB signal cascade. However, further investigations are needed to confirm how and when the high magnitude initiate to influence the inflammatory signaling network since the IL-1β-induced IKK activity was higher than that of high magnitude DCS treated sample in the presence of IL-1β at the earlier time point (Fig. 3C).

In addition to the changes in IκB-α, the degradation of IκB-β was examined, and the results showed similar trend observed in IκB-α degradation (Fig. 3G and 3H). IL-1β-induced degradation of IκB-β was attenuated by the application of 10% DCS while 30% DCS intensified its effect. Moreover, low magnitudes of DCS appeared to upregulate the protein synthesis as opposed to high magnitudes of DCS-induced degradation.

### DCS differentially control NF-κB nuclear translocation in a magnitude-dependent manner

**Figure 4:**
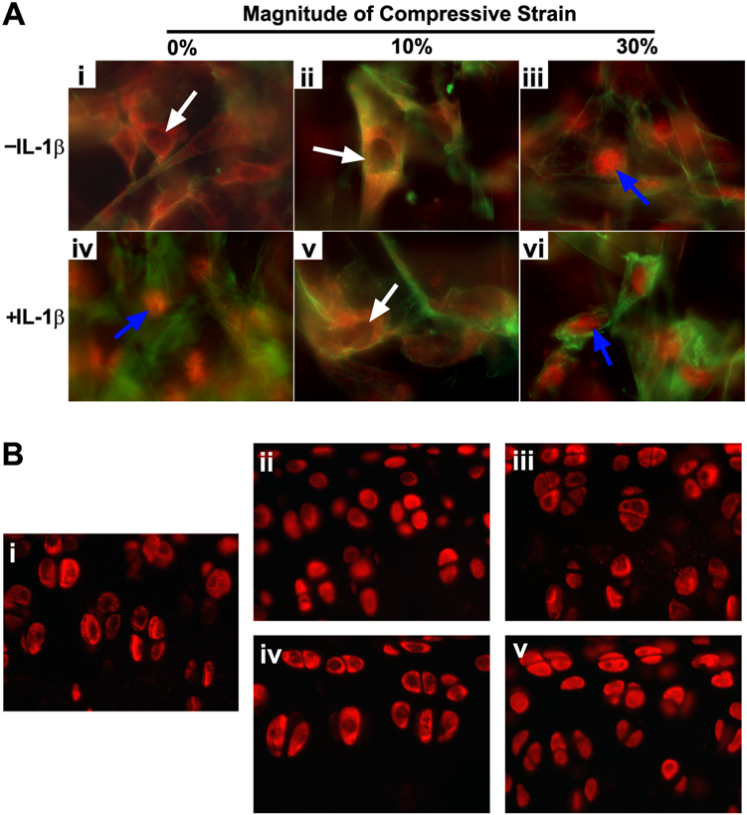
Regulation of NF-κB nuclear translocation by DCS. (A) Immunofluorescence analysis shows that DCS of low magnitudes (10%) inhibits IL-1β-induced NF-κB (p65, red staining) nuclear translocation; cells exposed to (i) no treatment, (ii) 10% DCS for 45 mins, (iii) 30% DCS for 45 mins, (iv) IL-1β (2 ng/ml) for 45 mins, (v) 10% DCS in the presence of IL-1β for 45 mins, and (vi) 30% DCS in the presence of IL-1β for 45 mins. Actin (green) is counter-stained by Phalloidin. (B) Immunohistochemical staining of NF-κB p65 (red) in chondrocytes present in the articular surface of the femurs showing the inhibition of NF-κB nuclear translocation by dynamic compression of physiological magnitudes and induction of NF-κB nuclear translocation by hyper-physiological loading; NF-κB (red) staining in cartilage exposed to (i) no treatment (control cartilage), (ii) IL-1β (5 ng/ml) incubation for 30 mins, (iii) IL-1β and dynamic compression of physiologic magnitudes (0.3 Hz, 3 times body weight) for 30 mins, (iv) dynamic compression of physiologic magnitudes alone, and (v) dynamic compression of high magnitude (0.1 Hz, 22 times body weight) for 10 times and subsequent incubation in medium for 30 mins.

NF-κB nuclear translocation, a signaling process downstream of IκB-α and IκB-β degradation, was investigated by immunofluorescence analysis (Fig. 4A). In the absence of IL-1β, 30% DCS applied for 45 minutes induced nuclear translocation of NF-κB as shown in the localized presence of p65 in the nuclei (Fig. 4Aiii), in contrast to its mostly cytoplasmic presence in control (Fig. 4Ai) or 10% DCS (Fig. 4Aii). As expected, the exogenously supplied IL-1β stimulated the NF-κB nuclear translocation evident in Figure 4Aiv. The IL-1β-induced nuclear translocation was inhibited by the applied DCS at 10% (Fig. 4Av) while it was not influenced by 30% DCS (Fig. 4Avi).

To examine the applicability of the *in vitro* observation to *in vivo*, rat femur explants were subjected to dynamic compression in the presence and absence of IL-1β. In these experiments, the explants were subjected to either physiological levels (3 times of body weight) or hyper-physiological levels (22 times of body weight) of dynamic compression in the absence or presence of IL-1β. The physiological levels of compression resulted in an average pressure of ∼400 KPa with a peak pressure of ∼600 KPa in the medial condyles, as analyzed by a piezoelectric sensor. For the hyper-physiological levels, an average pressure of ∼700 KPa with a peak pressure of ∼1800 KPa was recorded. The localization of NF-κB p65 in the sections prepared from the central region of medial condyles was immunofluorescently examined (Fig. 4B). Similar to the *in vitro* study, the cytoplasmic presence of most of NF-κB was observed in the untreated control (Fig. 4Bi), and nuclear translocation was induced by IL-1β based on the stain-filled nuclei (Fig. 4Bii). Less NF-κB nuclear localization was observed in either physiologically compressed sample in the presence (Fig. 4Biii) or absence (Fig. 4Biv) of IL-1β. However, hyper-physiological high magnitude compression alone induced NF-κB nuclear translocation (Fig. 4Bv).

### The mechanistic mathematical model helps understanding the experimental observations

**Figure 5:**
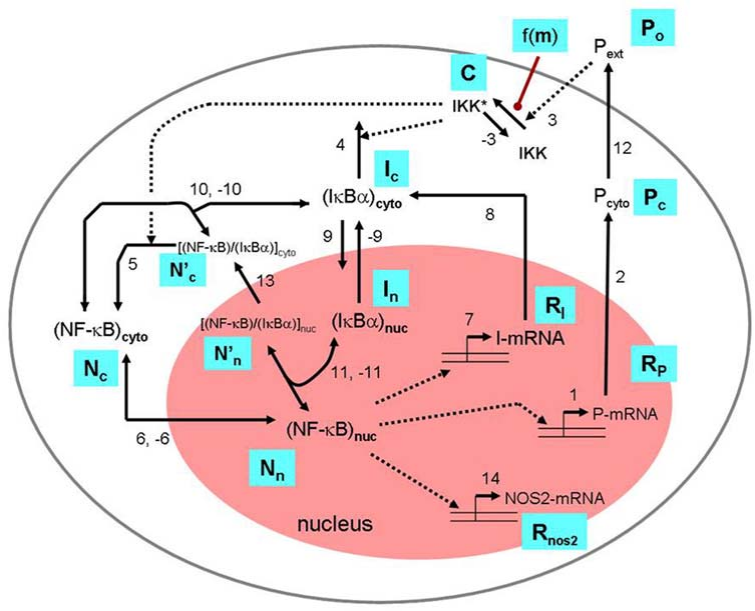
Proposed scheme explaining the magnitude-dependent regulation of NF-κB activity by a mathematical model. Steps considered in the mathematical model are from activation of IKK (step 3), which is regulated by a mechanical signal *m* in a manner described by the function *f(m)* that controls nuclear localization of NF-κB (step 6) and subsequent expression of pro-inflammatory mediators P_c_ (steps 1, 2), *NOS2* (step 14), and the protein IκB-α (steps 7, 8). Descriptions of the other steps are given in Table 1. The cell nucleus is colored pink and the periphery of the cell (plasma membrane) is indicated by the large grey oval. The symbols in blue are those used in the model equations in Tables 1 and 2. Note that the factor *b* in the production rate of the P_o_ (see third equation in Table 2) accounts for the contribution of other cells to this extracellular P_o_.

**Figure 6:**
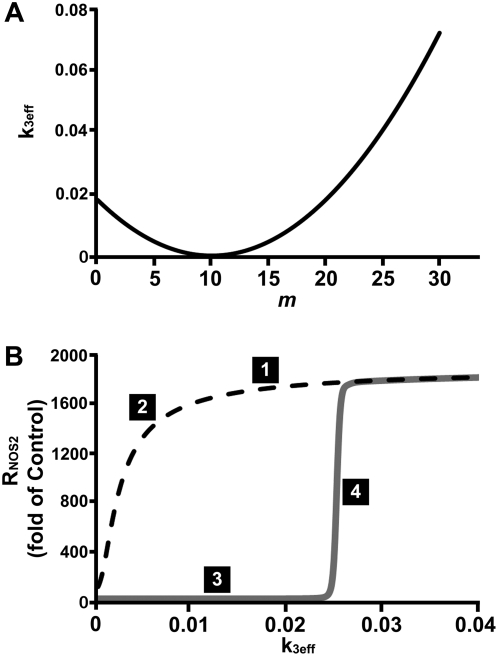
Mathematical model predictions of k_3eff_ and R_NOS2_. (A) Predicted k_3eff_ as a function of *m*. (B) Model prediction of the *steady states* of R_NOS2_ (NOS2 mRNA) as a function of k_3eff_. The solid curve was generated by increasing k_3eff_ slowly (with rate dk_3eff_/dt = 0.00001) and solving the set of differential equations in Table 2. The dotted curve was generated by decreasing k_3eff_ (with rate dk_3eff_/dt = −0.00001) starting from the endpoint of the solid curve. Location labeled 1 corresponds to the point on the curve of Figure 6A for *m* = 0 and k_3eff_∼0.018. Initially as *m* increases the value of k_3eff_ approaches zero, and this corresponds to the transition shown in the region 2 of the figure. As *m* increases further, hardly any change in R_NOS2_ is predicted (region 3 above) which corresponds to *m* between *m*_th2_ and *m*_th1_ in Figure 7A. Increasing *m* beyond *m*_th1_ leads to the transition from 3 to 4 corresponding to the sharp increase in R_NOS2_ (region 4 in the Fig. 7A). Initial values of parameters derived from the equilibrium state for P_0_ = 0 were R_P_ = 0.00944, P_c_ = 0.000925, P_0_ = 0.259127, C = 0.001337, R_I_ = 0.011025, I_c_ = 0.020399, I_n_ = 0.011686, N_c_ = 0.0000426, R_NOS2_ = 0.000616, N_n_ = 0.000647, N_pc_ = 0.042536, and N_pn_ = 0.000274.

It was shown previously that IKK is the key moleucule regulated by biomechanical signals [8], [10]. Low physiological magnitudes of biomechanical forces prevent phophorylation of TAK1 (TGF-β activating kinase), which in turn inhibits phosphorylation of IKK, resulting in attenuating further downstream NF-κB signaling cascade. Now we show that IKK activity, estimated from the phosphorylation of IκB-α, is regulated by DCS; initially it is suppressed as the magnitude of DCS increases and then increases again with further raising in magnitude (Fig. 3C and 3D). This observation of biphasic IKK activation is incorporated in the model of Figure 5 through the rate coefficient of activation of IKK, that is, *k*_3_f(*m*), where f(*m*) is a function of DCS magnitude (see Table 1). The factor *k*_3_f(*m*) is referred to as *k*_3eff_. A simple choice of the form of the function f(*m*) is the parabola *a*(*m*−*m*_0_)^2^, where *a* is set so that f(0) = 1, and *m*_0_ is set so that the observed threshold in the magnitude of DCS is approximated (Fig. 6A) A plot of R_NOS2_ (NOS2 mRNA) versus *k*_3eff_ in *steady states* is given in Figure 6B. These *steady states* are the long-term levels of R_NOS2_, and are determined by setting all the differential equations in Table 2 to zero.

**Table 1:** List of rate expressions and parameter values used in the model (symbols of molecules are given in Figure 5).

Description of Step	Rate Expressions, v	Parameter Values	Ref.
Cytokine transcription	v_1_ = k_1_N_n_^2^/(K_M1_+N_n_^2^)	k_1_ = 2.5 µM min^−1^	estimate
		K_M1_ = 0.1 µM^2^	estimate
Cytokine translation	v_2_ = k_2_R_p_	k_2_ = 0.5 min^−1^	estimate
Cytokine-induced activation of IKK, inhibited by mechanical signal m	v_3_ = k_3_P_o_(C_tot_−C)f(m)	k_3_ = 0.018 µM^−1^min^−1^	estimate
		C_tot_ = 0.03 µM	estimate
	f(m) = α(m−m_L_)^2^	m_L_ = 10	estimate
		α = (1/m_L_)^2^	estimate
Deactivation of IKK	v_−3_ = k_−3_C	k_−3_ = 0.1 min^−1^	estimate
IKK-induced degradation of IκB-α	v_4_ = k_4_CI_c_/(K_M4_+I_c_)	k_4_ = 0.0018 min^−1^	[18]
		K_M4_ = 0.0569 µM	[18]
IKK-induced degradation of IκB-α bound to NF-κB	v_5_ = k_5_CN^'^_c_/(K_M5_+N^'^_c_)	k_5_ = 0.36 min^−1^	[18]
		K_M5_ = 0.0392 µM	[18]
Nuclear localization of NF-κB	v_6_ = k_6_N_c_	k_6_ = 5.4 min^−1^	[18]
Nuclear export of NF-κB	v_−6_ = k_−6_N_n_	k_−6_ = 0.0048 min^−1^	[18]
I(B-( transcription	v7 = k7Nn2/(KM7+Nn2)	k7 = 0.1 µM min^−1^	estimate
		K_M7_ = 0.1 µM^2^	estimate
IκB-α translation	v_8_ = k_8_R_i_	k_8_ = 0.2448 min^−1^	[18]
Nuclear localization of IκB-α	v_9_ = k_9_I_c_	k_9_ = 0.018 min^−1^	[18]
Nuclear export of IκB-α	v_−9_ = k_−9_I_n_	k_−9_ = 0.012 min^−1^	[18]
Association of NF-κB with IκB-α in cytoplasm	v_10_ = k_10_N_c_I_c_	k_10_ = 30.0 µM^−1^min^−1^	[18]
Dissociation of NF-κB-IκB-α complex in cytoplasm	v_−10_ = k_−10_N^'^_c_	k_−10_ = 6.0×10^−5^ min^−1^	[18]
Association of NF-κB with IκB-α in nucleus	v_11_ = k_11_N_n_I_n_	k_11_ = 30.0 µM^−1^min^−1^	[18]
Dissociation of NF-κB-IκB-α complex in nucleus	v_−11_ = k_−11_N^'^_n_	k_−11_ = 6.0×10^−5^ min^−1^	[18]
Cell secretion of cytokines	v_12_ = k_12_P_c_	k_12_ = 5.0 min^−1^	estimate
Secretion by cell population	bv_12_	b = 1.12	estimate
Nuclear export of NF-κB-IκB-α complex	v_13_ = k_13_N^'^_n_	k_13_ = 0.828 min^−1^	[18]
NOS2 transcription	v_14_ = k_14_N_n_^2^/(K_M1_+N_n_^2^)	k_14_ = 2.5 µM min^−1^	estimate
Basal transcription of cytokine	a_1_	a_1_ = 1.5×10^−4^ µM min^−1^	[18]
Basal transcription of NOS2	a_3_	a_3_ = 0	estimate
Basal transcription of IκB-α	a_2_	a_2_ = 1.848×10^−4^ µM min^−1^	[18]
Constitutive degradation rates of R_p_	d_1_R_p_	d_1_ = 0.017 min^−1^	estimate
Constitutive degradation rates of P_c_	d_2_P_c_	d_2_ = 0.1 min^−1^	estimate
Constitutive degradation rates of P_o_	d_3_P_o_	d_3_ = 0.02 min^−1^	estimate
Constitutive degradation rates of R_i_	d_4_R_i_	d_4_ = 0.0168 min^−1^	[18]
Constitutive degradation rates of I_c_	d_5_I_c_	d_5_ = 0.12 min^−1^	[18]
Constitutive degradation rates of R_nos2_	d_6_R_nos2_	d_6_ = 0.017 min^−1^	estimate

**Figure 7:**
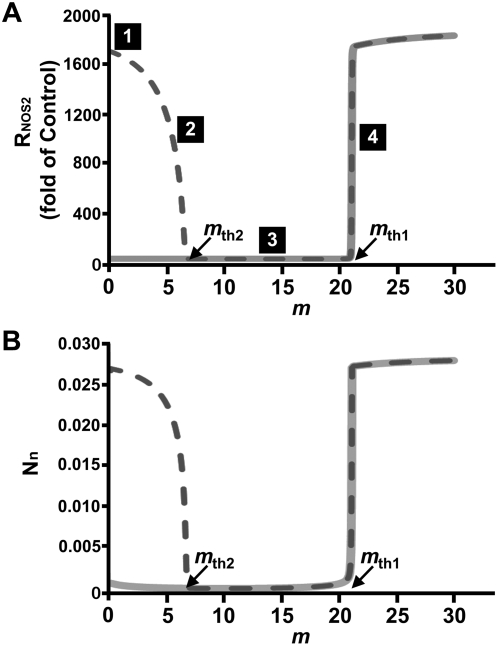
Mathematical model predictions of R_NOS2_ and NF-κB nuclear concentration. Predicted (A) R_NOS2_ and (B) NF-κB concentration in nucleus (N_n_) with respect to magnitude of biomechanical stimulation (*m*) in the absence (P_0_ = 0, solid lines) or presence (P_0_ = 25, dotted lines) of inflammatory cytokine at *steady states*. Initial values of parameters derived from the equilibrium state for P_0_ = 0 were R_P_ = 0.00944, P_c_ = 0.000925, P_0_ = 0.259127, C = 0.001337, R_I_ = 0.011025, I_c_ = 0.020399, I_n_ = 0.011686, N_c_ = 0.0000426, R_NOS2_ = 0.000616, N_n_ = 0.000647, N_pc_ = 0.042536, and N_pn_ = 0.000274; for P_0_ = 25: R_P_ = 1.06679, P_c_ = 0.104587, P_0_ = 29.2808, C = 0.0.0252157, R_I_ = 0.053822, I_c_ = 0.0892936, I_n_ = 0.00196127, N_c_ = 0.000317215, R_NOS2_ = 1.05796, N_n_ = 0.0269191, N_pc_ = 0.0143506, and N_pn_ = 0.00191274.

The two lines in Figure 6B indicate that the system exhibits bistability for k_3eff_ between 0 and ∼0.024. This phenomenon is characterized by having two stable steady states that coexist for a given set of parameters. The solid curve of Figure 6B explains the origin of the threshold (at *m* = *m*_th1_) observed in the experiment with no initial addition of IL-1β (Fig. 2, solid line), as well as the origin of another threshold *m*_th2_ (*m*_th2_<*m*_th1_), which could not be clearly observed in the experiment. The predicted *m*_th2_ is the point where expression of pro-inflammatory gene (*NOS2*) is completely suppressed in the presence of inflammatory cytokine (Fig. 7A). At *k*_3eff_∼0.024, corresponding to *m* = *m*_th1_, synthesis of R_NOS2_ turns on. At *k*_3,eff_ = 0, corresponding to *m* = *m*_th2_, synthesis of R_NOS2_ turns off. These two model-predicted thresholds are shown explicitly in Figure 7A. In Figure 7B, the corresponding levels of nuclear NF-κB concentration (N_n_) in *steady state* are shown. The model predicts that the nuclear localization of NF-κB parallels that of the synthesis of R_NOS2_ which is a target of active NF-κB as experimentally observed [14].

**Table 2:** Differential equations used for the Model (the rate expressions v_i_’s are defined in Table 1).

dR_p_/dt = a_1_+v_1_−d_1_R_p_
dP_c_/dt = v_2_−v_12_−d_2_P_c_
dP_o_/dt = bv_12_−d_3_P_o_
dC/dt = v_3_−v_−3_
dR_i_/dt = a_2_+v_7_−d_4_R_i_
dI_c_/dt = v_8_+v_−9_+v_−10_−(v_9_+v_4_+v_10_)−d_5_I_c_
dI_n_/dt = v_9_+v_−11_−(v_−9_+v_11_)
dN_c_/dt = v_−10_+v_−6_+v_5_−(v_10_+v_6_)
dN_n_/dt = v_6_+v_−11_−(v_11_+v_−6_)
dN^'^_c_/dt = v_10_+v_13_−(v_−10_+v_5_)
dN^'^_n_/dt = v_11_−(v_−11_+v_13_)
dR_nos2_/dt = a_3_+v_14_−d_6_R_nos2_

Note that the computer simulations show an identical threshold (*m*_th1_) for the cases with (dotted line) and without IL-1β (solid line) in Figure 7A. However, the model does not predict the accurate levels of R_NOS2_ observed in the experiment (Figure 2). This discrepancy is expected since the experimental system did not reach an equilibrium state. Furthermore, many simplifying assumptions of the model such as ignoring the hundreds of NF-κB targets that affect *NOS2* expression as well as simultaneous activation of other signaling pathways involved in the regulation of *NOS2* expression likely affect the equilibrium state of NF-κB [15], [16], [17].

In summary, we present an experimental evidence-based mathematical model that provides insight into the regulation of pro-inflammatory gene by dynamic compressive forces in chondrocytes. The data demonstrates that the magnitude of biomechanical signals perceived by the cells is the critical event that controls the activation or inhibition of pro-inflammatory gene induction, in the presence or absence of an inflammatory stimulus. Furthermore, there exists a threshold in the magnitude of DCS for inducing pro-inflammatory genes even without induction by IL-1β; below this threshold, *NOS2* expression is not observed. In the presence of IL-1β, we showed that increasing magnitudes of DCS decreases *NOS2* production, but only if the DCS magnitude is less than the aforementioned threshold where *NOS2* expression begins to increase again. It is interesting to note that the induction of *NOS2* synthesis occurs at the same threshold of DCS magnitude, with or without IL-1β.

We further showed that the NF-κB pathway is involved in the transduction of the mechanical signals generated by DCS. Evidence was presented that the direct interface between mechanical signals and this pathway is IKK, and that the activity of IKK is biphasic (first decreasing and then increasing with increasing DCS magnitude). Using a comprehensive mechanistic model of the NF-κB network and the dependence of IKK activity with the magnitudes of DCS, we were able to explain the existence of the threshold, labeled as m_th1_ in Figure 7. This threshold arises due to the bistable behavior of the networks originating from the positive feedback loop between NF-κB and its target genes such as *IL-1β*, *TNF-α* and *NOS2*. Interestingly, this model also predicts another threshold shown as *m_th2_* in Figure 7, above which (and below *m_th1_*) cytokine-induced *NOS2* synthesis is suppressed. This novel model lays the groundwork to explain how biomechanical signals by gentle exercise may inhibit inflammation and restore cartilage, and how at high magnitudes become inflammatory and initiate destruction of the cartilage.

## Materials and Methods

### Cell culture in 3-D scaffolds

Mesenchymal cell line, C3H10T1/2 cells (ATCC) were expanded in monolayer with a growth medium (DMEM (Gibco), 10% FBS, 10 µg/ml penicillin, 100 U/ml streptomycin, 2 mM L-glutamine). Subsequently, approximately 600,000 cells/scaffold were seeded into well-characterized electrospun poly(ε-caprolactone) scaffolds having 6 mm in diameter and 3 mm in thickness, as described previously [12]. The cells in the scaffolds were cultured for 48 hours in a differentiation medium (Ham's F-12 (Gibco), 10% FBS, 10 ug/ml penicillin, 100 U/ml streptomycin, 2 mM L-glutamine) prior to subjecting to various conditions. Chondrocytic differentiation of the cells in the scaffolds were confirmed by examining the synthesis of glycosaminoglycan using Alcian blue staining and chondrogenic/chondrocytic gene/protein expression using real-time reverse transcription polymerase chain reaction (rt-PCR) and Western blot (Figure S1).

### Application of DCS

Following 48 hours of cultivation, the cell-scaffold constructs were subjected to various magnitudes of DCS at 1 Hz in the absence or presence of recombinant human IL-1β (2 ng/ml; Calbiochem) using a custom-made compression device [12]. The regimens used in this study are shown in Figure 1. For *NOS2* and *TNF-α* gene expression analysis, 0%, 7.5%, 15%, 22.5% or 30% of DCS was used. After observing the magnitude-dependent inflammatory gene regulation by biomechanical signals, 0%, 10% (representative regimen for anti-inflammatory magnitude) or 30% (representative regimen for pro-inflammatory magnitude) of DCS was used for protein and immunoflurescence analysis to confirm the immunomodulation through the NF-κB signal transduction.

### Analysis of mRNA expression by rt-PCR

After the application of DCS, pro-inflammatory agent or combination of both for 45 minutes, the samples were further incubated for 75 minutes. Total RNA pooled from two samples per condition was subsequently extracted using the RNeasy micro kit as recommended (Qiagen). Total RNA (1 µg) was measured using the Nanodrop spectrophotometer (ND-1000, Nanodrop tech.) and first strand synthesis performed using the Superscript III Reverse Transcriptase Kit (Invitrogen). Custom-designed gene-specific primers were used to perform real time-PCR using the Bio-Rad SYBR Green Master Mix (Bio-Rad) in the iCycler iQ Real-Time PCR System (Bio-Rad). Similarly, total RNAs from monolayer-cultured and scaffold-cultured cells without any treatments were extracted to examine the chondrocytic differentiation of C3H10T1/2 cells, and cDNA synthesized as previously described. Following primers were used: *NOS2*-sense 5′-CACCAAGCTGAACTTGAGCGA-3′, *NOS2*-anti-sense 5′-GCCCCATAGGAAAAGACTGCA-3′; *TNF-α*-sense 5′- AGAGGCACTCCCCCAAAAGAT-3′, *TNF-α*-anti-sense 5′- TGCCACAAGCAGGAATGAGA-3′; *RPS18*-sense 5′-GGAAAATAGCCTTCGCCATCACT-3′, *RPS18*-anti-sense 5′-GCCAGTGGTCTTGGTGTGCTGAC-3′; *WNT5A*-sense 5′-GGCATCAAGGAATGCCAGTA-3′, *WNT5A*-anti-sense 5′-GTACGTGAAGGCCGTCTCTC-3′; *SOX9*-sense 5′-AACGGCTCCACTCAAGAACAAG-3′, *SOX9*-anti-sense 5′-TCCGTTCTTCACCGACTTCCTC-3′; *ACAN*-sense 5′-ACTGTCAAAGCACCATGCCTT-3′, *ACAN*-anti-sense 5′-CCATTCAGTCTGTTTTCTTGCC; *COL2A1*-sense 5′-AAACTGGTGGAGCAGCAAGAG-3′, *COL2A1*-anti-sense 5′-ATCTGGACGTTAGCGGTGTTG-3′. The thermocycling protocol was: 95°C for 3 min, 40 cycles of denaturation at 95°C for 30 s, annealing at 60°C for 30 s, and extension at 72°C for 30 s. Collected data were analyzed by the comparative threshold cycle (C_T_) method using RPS18 expression as an endogenous normalization control.

### Protein phosphorylation and degradation

Activation and degradation of proteins in the NF-κB signaling pathway were analyzed by Western blot after the application of DCS in the absence or presence of IL-1β for 15 or 30 minutes as indicated in Figure 1. Total cell extracts (40 µg/lane) were separated by SDS-10% PAGE and then electrophoretically transferred onto nitrocellulose membrane (Bio-Rad). The blots were subsequently probed with anti-phospho-ser32/36-IκB-α (Cell Signaling), anti-IκB-α and anti-IκB-β (Santa Cruz Biotechnology). The bound primary antibodies were detected by either IR-Dye 680 or 800 conjugated secondary antibodies (LI-COR Biosciences). To normalize protein loading, the membranes were probed with anti-β-actin (Sigma-Aldrich) and detected by IR-Dye labeled secondary antibodies. The bands were densitometrically analyzed by LiCor Odyssey imaging system (LiCor).

### Immunofluorescence

Following the treatments of the cell-scaffold constructs as indicated in Figure 1, the samples were fixed with 2% paraformaldehyde. The fixed constructs were horizontally cut in the middle followed by cell permeabilization with 0.2% Triton-X for 30 minutes. Cells were then stained with anti-p65 primary antibody (Santa Cruz Biotechnology) and CY3-conjugated secondary antibody (Jackson ImmunoResearch Laboratories). To reveal the morphology of the cells, F-actin was subsequently stained with fluorescein isothiocyanate (FITC) labeled phalloidin (Sigma-Aldrich). The stained samples were observed under an epifluorescence microscope (Axioplan2; Carl Zeiss).

### Compression of femur explants and immunohistochemical analysis

The femur explants were obtained from 10–12-week-old female Sprague-Dawley rats (Harlan) and kept in ice-cold PBS. Prior to experiments, the explants were equilibrated to 37°C in serum-free culture medium (Ham's F-12, 10 µg/ml penicillin, 100 U/ml streptomycin). A computer controlled load frame (Model 1000R12; TestResources) equipped with an electronically calibrated 50 lb load cell (Model SM-50; TestResources) was used. Using load control, condyle-side of rat femur held by a custom-made fixture was compressed against a ½ inch-thick ultra-soft polyurethane sheet in various conditions including dynamic compression of approximately 3 times of body weight (2 lbs) at 0.5 Hz for 30 minutes, or approximately 22 times of body weight (15 lbs) at 0.1 Hz for 10 times followed by 30 minutes of incubation, in the absence or presence of IL-1β (5 ng/ml) in serum-free culture medium. A computerized contact area and pressure measurement system, K-Scan 4000 (Tekscan) was placed between the rat femur and the polyurethane sheet to assess differential peak pressure with respect to contact area. The peak pressure per area was calculated by the captured image analysis. Each condition was performed three times with femurs obtained from different animals.

Following the application of dynamic compression and pressure measurements, the samples were fixed in 10% formalin solution. The fixed samples were decalcified using Cal-EX (Fisher Scientific), and embedded in paraffin for sectioning. The sections of medial condyles were re-hydrated using xylene and ethanol-water series, then subsequently stained with NF-κB p65 primary antibody (Santa Cruz Biotechnology) and CY3-conjugaed secondary antibody (Jackson ImmunoResearch Laboratories). The stained sections were observed under an epifluorescence microscope.

### Statistical Analysis

At least two independent experiments were performed and the most representative data has been presented. For phenotypic characterization of the cells, T-test was used to determine statistical significance (*; p≤0.05, **; p≤0.01) in comparisons made between the monolayer-cultured and the 3-D scaffold-cultured cells.

### Model of the NF-κB network with mechanical signaling

The mechanistic steps comprising the model NF-κB network are shown in Figure 5. Many of the steps are similar to a model of the Hoffmann group [18]; the essential additions are the steps for the production of cytokine (P_c_ and P_o_) and the effect of mechanical signal (represented by a function *f(m)* of the mechanical load *m*) on the activation of IKK. Description of the individual steps and their rate expressions, and the parameter values used are listed in Table 1. The differential equations that describe the dynamics of the system are given in Table 2. An essential feature of the model is the function *f(m)* which incorporates experimental observations on how the magnitude of mechanical signals affect the activity of the enzyme IKK. The form of the function *f(m)* used in Table 1 encodes our observation that, as *m* increases, inflammatory products is initially inhibited in the presence of exogenous cytokine (IL-1β) but increases after reaching a certain threshold value of *m*. It is not yet known exactly how mechanical signaling interfaces with the molecular network shown in Figure 5. The set of ordinary differential equations in Table 2 is numerically solved using the software *BerkeleyMadonna* (www.berkeleymadonna.com).

## Supporting Information

Figure S1 Chondrogenesis of mesenchymal cells in 3-D culture. C3H10T1/2 cells were cultured in electrospun scaffolds for 48 hours and the proteoglycan synthesis was assessed by Alcian blue staining (A), gene expression by real time RT-PCR (B), and protein expression by Western blot (C). (A) The cell-scaffold constructs showed the presence of proteoglycan synthesis on the top surface, and in cross sections through 1/4, 1/2 and 3/4 height of the scaffolds confirming significant deposition of extracellular matrix. (B) chondrogenic (*WNT5A* and *SOX9*) and chondrocytic (*ACAN* and *COL2*) gene expression in cells grown in the scaffolds (black bars) compared to those grown in the tissue culture dishes (gray bars) confirming the chondrocytic differentiation of the cells by 3-D culture (n = 6, *; *p*<0.05, **; *p*<0.01). (C) Aggrecan, collagen type II and type I protein expression for the cells grown in the tissue culture dishes (left lane, monolayer) and in the scaffolds (right lane, scaffold) (representative gels out of three separate experiments are shown); scaffold-cultured cells expressed aggrecan and collagen type II proteins while no expression of those was observed in the tissue culture dish grown cells. In addition, collagen type I protein expression was substantially suppressed in the scaffold-cultured cells.
(4.51 MB TIF)
