# Supplementary figures and images for: Biomechanical Thresholds Regulate Inflammation through the NF-κB Pathway: Experiments and Modeling
Source: PLoS One. 2009 Apr 16;4(4):e5262. doi: 10.1371/journal.pone.0005262 (PMC2667254; doi:10.1371/journal.pone.0005262)

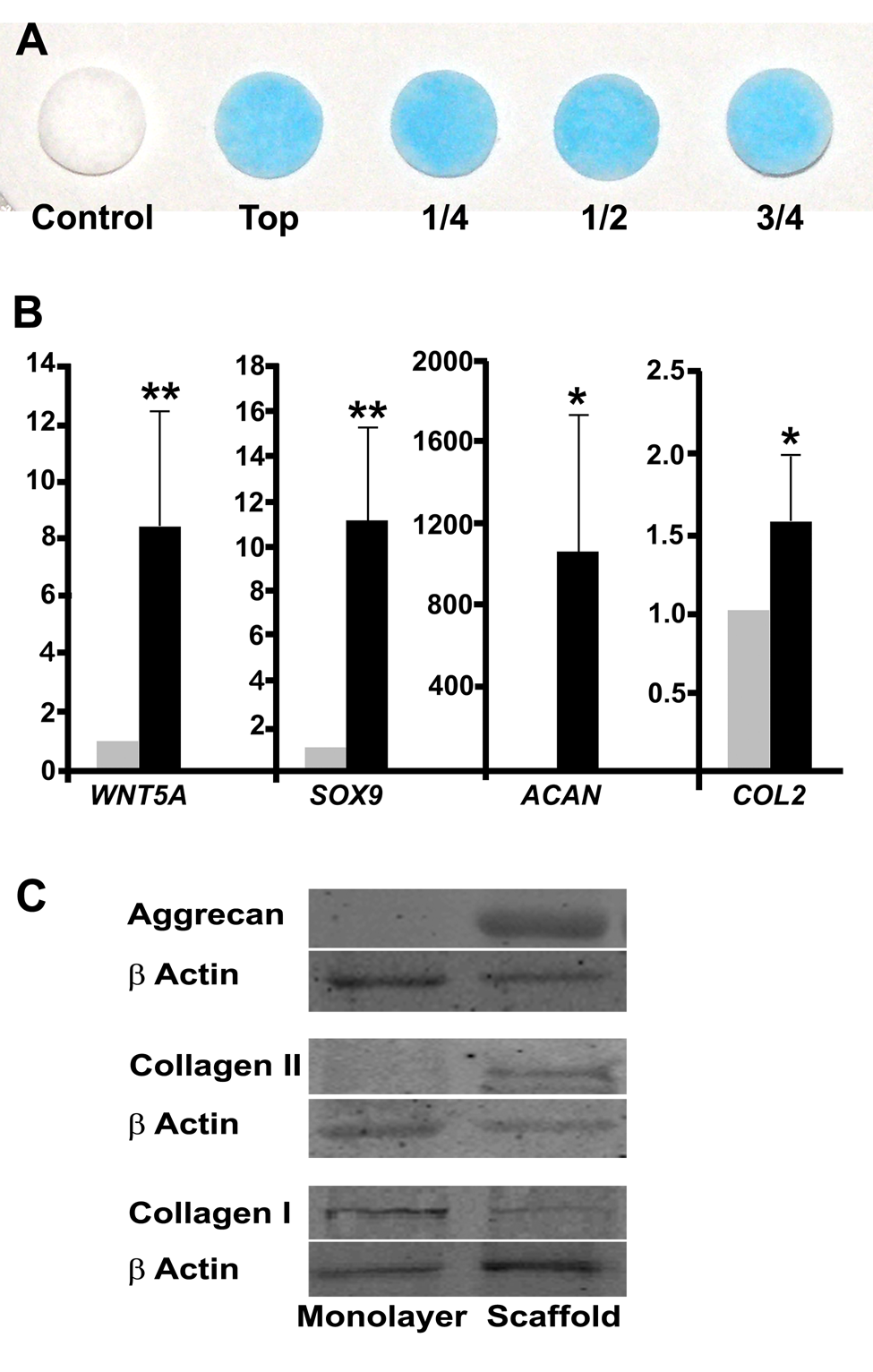

Supplement: Figure S1 — Chondrogenesis of mesenchymal cells in 3-D culture. C3H10T1/2 cells were cultured in electrospun scaffolds for 48 hours and the proteoglycan synthesis was assessed by Alcian blue staining (A), gene expression by real time RT-PCR (B), and protein expression by Western blot (C). (A) The cell-scaffold constructs showed the presence of proteoglycan synthesis on the top surface, and in cross sections through 1/4, 1/2 and 3/4 height of the scaffolds confirming significant deposition of extracellular matrix. (B) chondrogenic (WNT5A and SOX9) and chondrocytic (ACAN and COL2) gene expression in cells grown in the scaffolds (black bars) compared to those grown in the tissue culture dishes (gray bars) confirming the chondrocytic differentiation of the cells by 3-D culture (n = 6, *; p<0.05, **; p<0.01). (C) Aggrecan, collagen type II and type I protein expression for the cells grown in the tissue culture dishes (left lane, monolayer) and in the scaffolds (right lane, scaffold) (representative gels out of three separate experiments are shown); scaffold-cultured cells expressed aggrecan and collagen type II proteins while no expression of those was observed in the tissue culture dish grown cells. In addition, collagen type I protein expression was substantially suppressed in the scaffold-cultured cells. (4.51 MB TIF) [file pone.0005262.s001.tif]
